# Axial compression performance of partially encased concrete columns with web opening

**DOI:** 10.1038/s41598-024-62632-9

**Published:** 2024-05-24

**Authors:** Jiongfeng Liang, Yunchen Wang, Wanjie Zou, Caisen Wang, Wei Li

**Affiliations:** 1 Faculty of Civil and Architecture Engineering, East China University of Technology, Nanchang, China; 2 College of Civil and Architecture Engineering, Guangxi University of Science and Technology, Liuzhou, China; 3 Faculty of Architecture, Civil and Transportation Engineering, Beijing University of Technology, Beijing, China; 4 College of Civil and Architecture Engineering, Wenzhou University, Wenzhou, China; 5 Key Laboratory of Engineering and Technology for Soft Soil Foundation and Tideland Reclamation of Zhejiang Province, Wenzhou, China

**Keywords:** Web opening, Opening shape, Opening rate, Partial buckling, Load-carrying capacity, Civil engineering, Mechanical engineering

## Abstract

The objective of this study is to comprehensively assess the behavior of partially encased concrete (PEC) columns with web openings under axial compression. The primary objectives of this study are to analyze damage patterns and investigate the influence of key parameters, such as concrete strength, opening rate, and opening shape, on the ductility index and ultimate load-carrying capacity. The study employs experimental testing to examine the response of the PEC columns, with a particular focus on the mechanisms of concrete fracture and flange flexing. Notably, the study reveals a significant impact of the opening rate on the bearing capacity, while the effect of opening shape is comparatively minor. Furthermore, computational analyses are conducted to deepen the understanding of structural behavior. The study builds upon existing research to propose a novel method for calculating the bearing capacity of PEC columns with web openings. This method introduces two discount factors to enhance predictive accuracy.

## Introduction

Columns serve as vital structural elements in building construction, providing essential support and stability. Among various column forms, steel–concrete structures have gained favor among engineers due to their superior mechanical properties^1–3^. A notable variation within this category is the web-opened partially encased concrete (PEC) column, which offers several advantages over traditional reinforced concrete components. This column type combines the strength of steel and concrete while incorporating web openings, resulting in reduced deadweight, improved fire resistance, savings in formwork, and accelerated construction timelines^4–8^. Moreover, in specialized applications, such columns can accommodate pipeline passages, further expanding their utility and potential for development.

Nowadays, engineers were increasingly studying the various properties of PEC components. Chicoine et al.^9^ identified the failure mechanisms of PEC columns, attributing damage to concrete cracking and local flange buckling. Further investigations by Kong et al.^10^ categorized damage modes into symmetric and opposing buckling, shedding light on the column's behavior under axial compression. Begum et al.^11^ simulated PEC columns, revealing fragile damage patterns in high-strength concrete infills. Song et al.^12^ conducted finite element analyses on various PEC column parameters, proposing stress prediction models for flange design. Qian et al.^4^ explored lightweight aggregate concrete applications, observing damage mechanisms akin to traditional concrete, such as spalling and flange buckling. Bian et al.^13^ focused on thin walled steel PEC columns, deriving axial load capacity calculations. Wang et al.^14^ investigated beam-column connections with PEC columns, evaluating axial bearing capacities at different web hole locations. Begum et al.^15^ simulated PEC composite columns with comparable steel sections, while Pham et al.^16^ tested circular and square web openings, highlighting stress concentration effects. Ritchie et al.^17^ examined buckling behavior in plates with central circular holes, contributing to understanding structural responses. Tao et al.^18^ expanded research to flexural behavior in thin-walled steel plates with openings, complementing Moen et al.^19^ findings on cold-formed steel members. Similarly, investigations by others^20–22^ emphasized the impact of different opening shapes on ultimate load-bearing capacity in short columns.

Despite the extensive studies that have been conducted, there remains a need for a comprehensive understanding of the axial compressive behavior of PEC columns with web openings. The objective of this experimental study is to analyze the effects of concrete strength, opening rate, and shape on the axial load-carrying capacity. Furthermore, a simplified design formula for PEC composite columns with web openings will be proposed in order to address existing research gaps and enhance structural design methodologies.

## Experimental program

### Design of specimens

**Figure 1:**
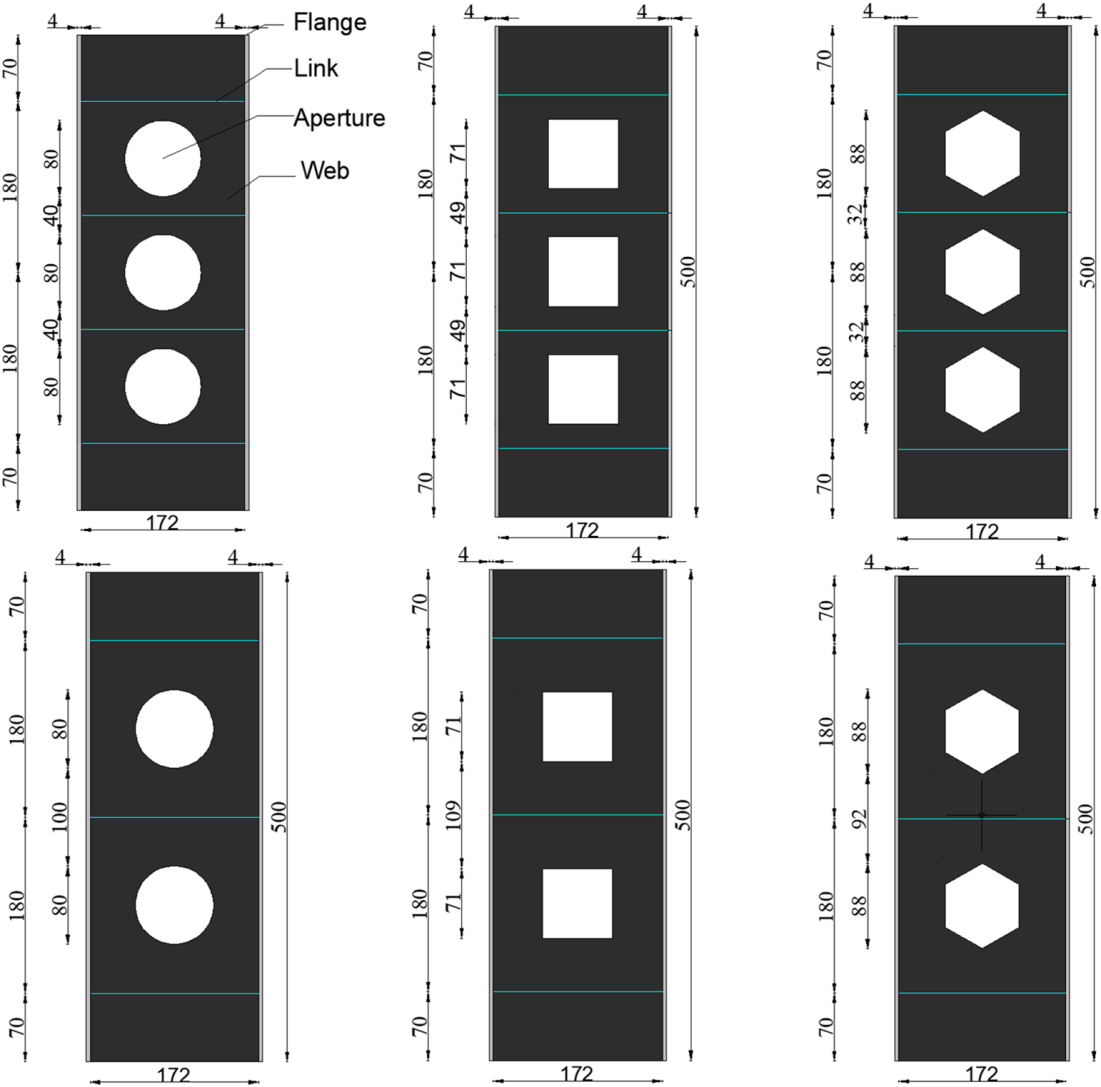
Location of specimen holes.

Within the context of this experiment, 8 stub columns were designed, among which 7 were PEC columns with web opening and 1 PEC column without web opening. A trade-off was made between economy and structure based on the research of previous scholars^5,6^. Dimensions were selected to meet the performance requirements of the design while reducing the cost as much as possible. All the specimens were 500 mm in length, and the thickness of the web and flanges is 4 mm. The parameters are determined to the concrete strength (C30, C40), the open hole rate (11.7% corresponds to 2 holes, and 17.5% corresponds to 3 holes), and the open hole shapes (circular, hexagonal, square). The size and dimensions of the openings are designed according to GB50017-2017^23^, and the opening rate is defined as the ratio of web opening area to total web area. In addition, the information of the test specimens are summarized in Table 1, and the locations of the specimen’ holes were shown in Fig. 1.

**Table 1:** The information of the tested specimen.

Specimens number	Specimen height (mm)	Section size H × B × t_w_ × t_f_	Concrete strength	Hole shape	Opening rate
PEC-30-11.7-C	500	180 × 180 × 4 × 4	C30	Circular	11.7%
PEC-40-11.7-C	500	180 × 180 × 4 × 4	C40	Circular	11.7%
PEC-30-11.7-H	500	180 × 180 × 4 × 4	C30	Hexagonal	11.7%
PEC-30-11.7-S	500	180 × 180 × 4 × 4	C30	Square	11.7%
PEC-30-17.5-C	500	180 × 180 × 4 × 4	C30	Circular	17.5%
PEC-30-17.5-S	500	180 × 180 × 4 × 4	C30	Square	17.5%
PEC-30-17.5-H	500	180 × 180 × 4 × 4	C30	Hexagonal	17.5%
PEC-30	500	180 × 180 × 4 × 4	C30	Solid	0

Where C stands for a circular hole, H stands for a hexagonal hole, and S stands for a square hole.

### Fabrication process of specimens

**Figure 2:**
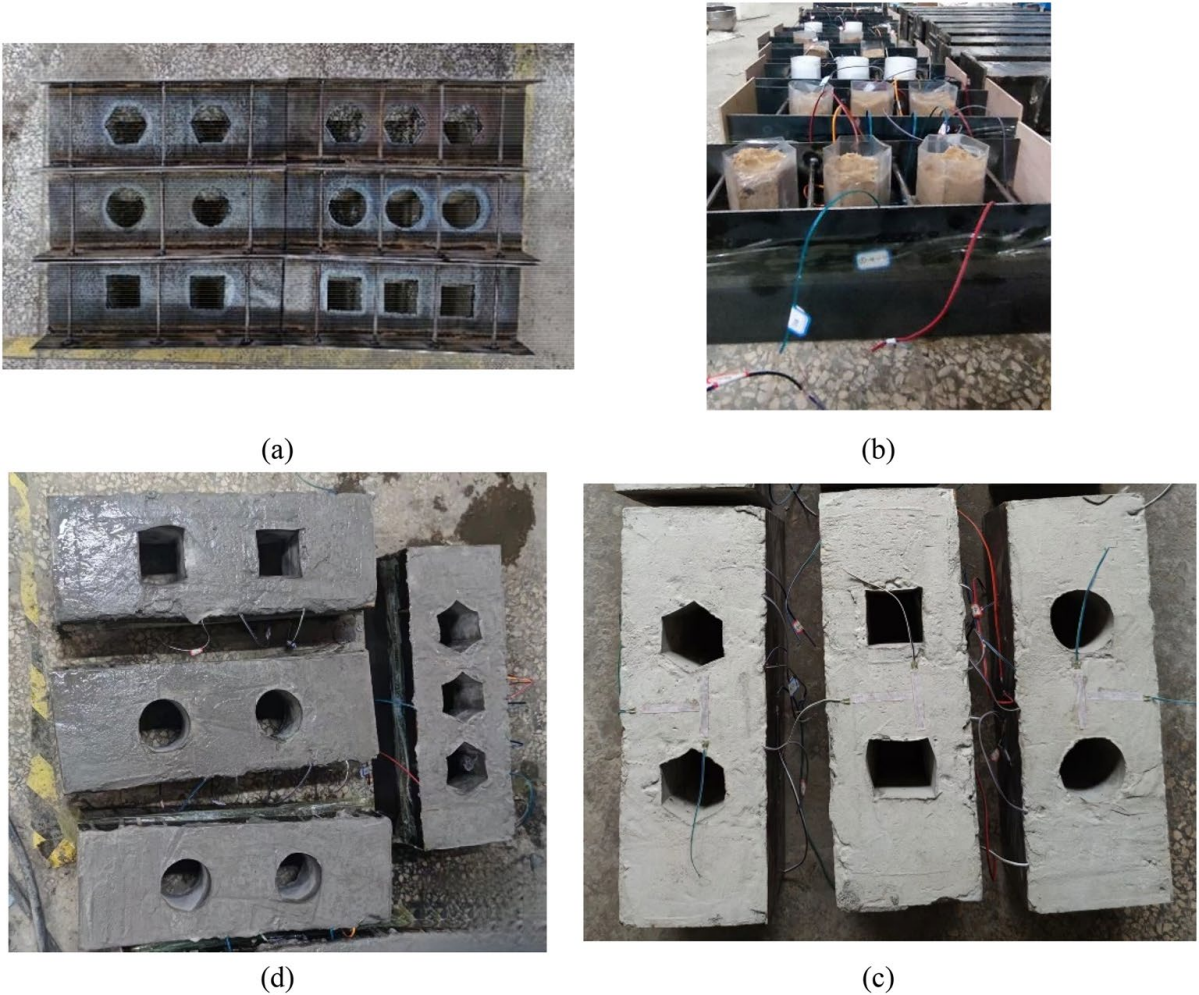
Specimen fabrication.

The flange and web plate were connected by welding. Oxygen welding was employed to create openings in the web of the H-shaped steel section according to predetermined scribed positions, as illustrated in Fig. 2a. Connecting links were welded at both ends of the flange after completing the openings. Subsequently, the left and right sides of the H-shaped steel section were secured with wooden boards to prevent concrete outflow. The openings were then filled with fine sand to prevent deformation, as shown in Fig. 2b. Finally, the concrete was poured in layers. One side of the H-shaped steel section was poured, and 48 h were allowed to elapse before turning the specimen over and pouring the other side. The structure was maintained for 28 days after the completion of pouring. The fabrication process of specimens is described in Fig. 2.

### Features of the material

#### Steel properties

**Figure 3:**
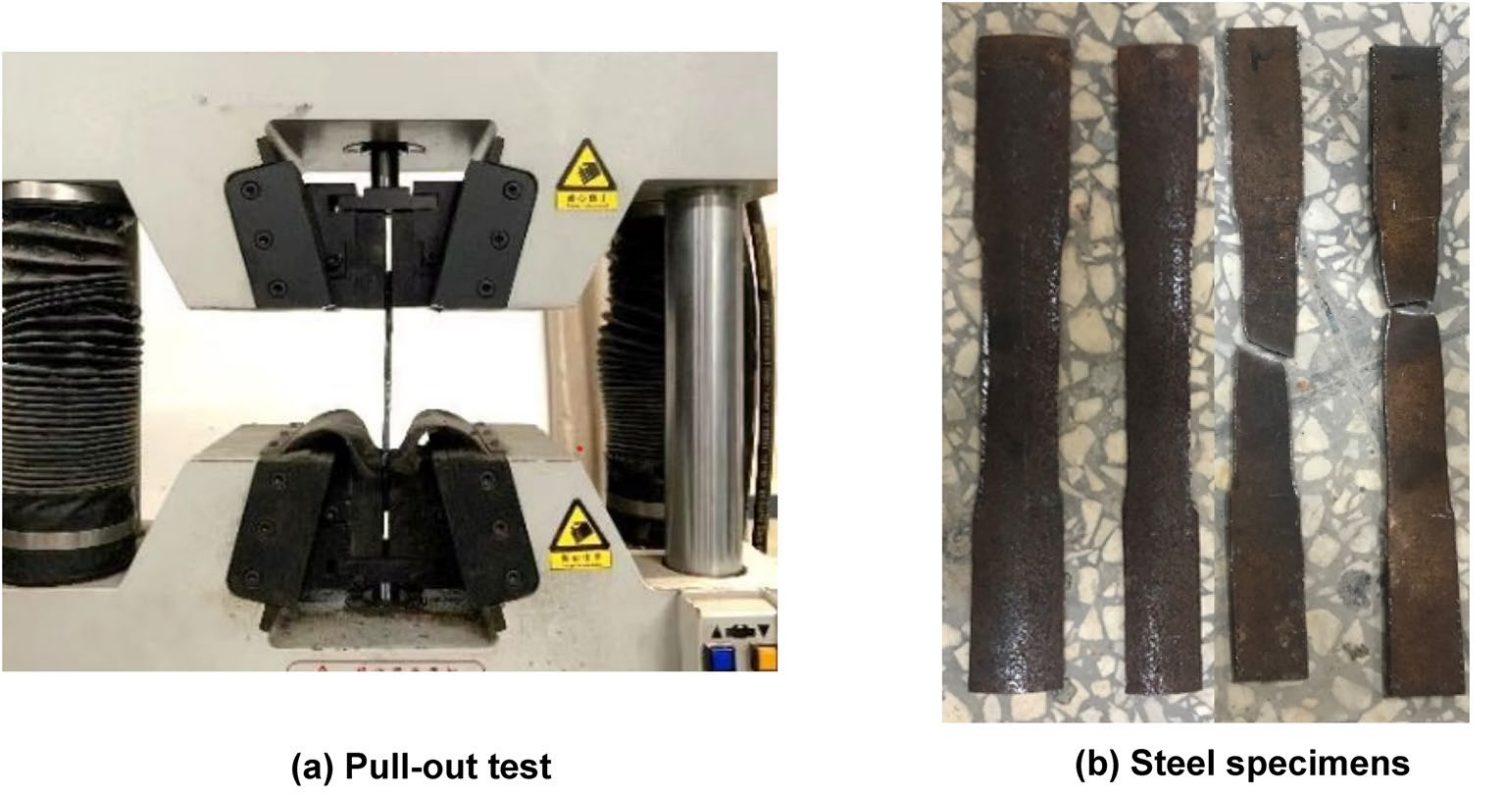
Steel testing.

To determine the characteristics of the steel, as shown in Fig. 3 the steel was placed in a testing machine to be tested^24^. The material properties of the steel are shown in Table 2, the yield strength of the link is 324 MPa with a diameter of 6 mm, where *f*_y_ is the tensile yield stress; *f*_u_ is the ultimate tensile stress; *E*_s_ is the elastic modulus.

**Table 2:** Material properties of steel.

Steel pattern	*f*_*y*_ (MPa)	*f*_u_ (MPa)	*E*_*s*_ (× 10^5^ MPa)
Flange	346	443	2.05
Web	352	437	2.03

#### Concrete properties

**Figure 4:**
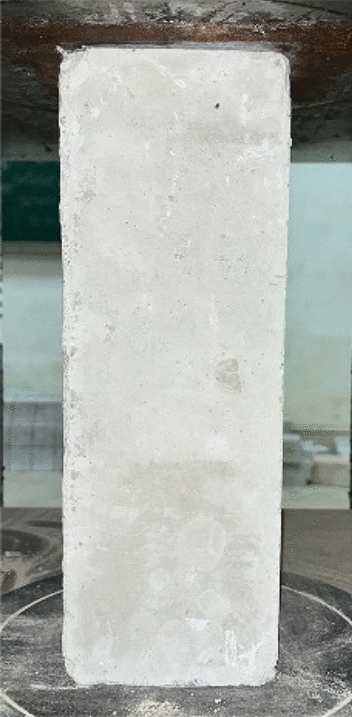
Initial loading.

**Figure 5:**
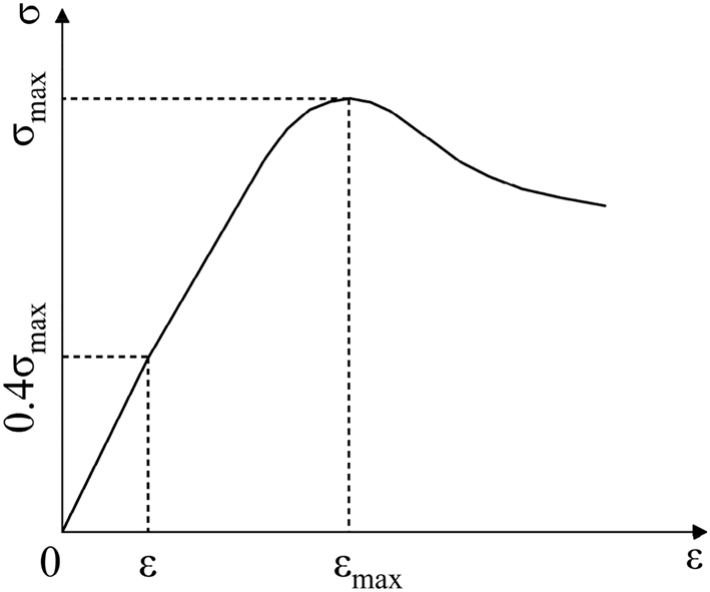
Standard concrete stress–strain curve.

This test used two strength levels of concrete: C30 and C40, and the mix proportions of the concrete are presented in Table 3. The compressive strength is determined according to the relevant test standards and methods^25^. The test results are shown in Table 3, which *f*_cu_ is the cubic compressive strength of concrete, *E*_c_ is the elastic modulus of concrete. Using 100 × 100 × 300 non-standard prismatic test blocks as shown in Fig. 4, the stress–strain curve obtained after axial compression load test, according to the origin to 0.4 times the peak stress point of the slope of the line derived from the modulus of elasticity, as illustrated in Fig. 5.

**Table 3:** Concrete proportioning and properties.

Grade of concrete strength	Coarse aggregate (Kg/m^3^)	Sand (Kg/m^3^)	Cement (Kg/m^3^)	Water (Kg/m^3^)	*f*_cu_ (MPa)	*E*_c_ (MPa)
C30	1187	533	446	205	36.7	3.01 × 10^4^
C40	1188	558	419	205	42.8	3.22 × 10^4^

### Installation and measurements

**Figure 6:**
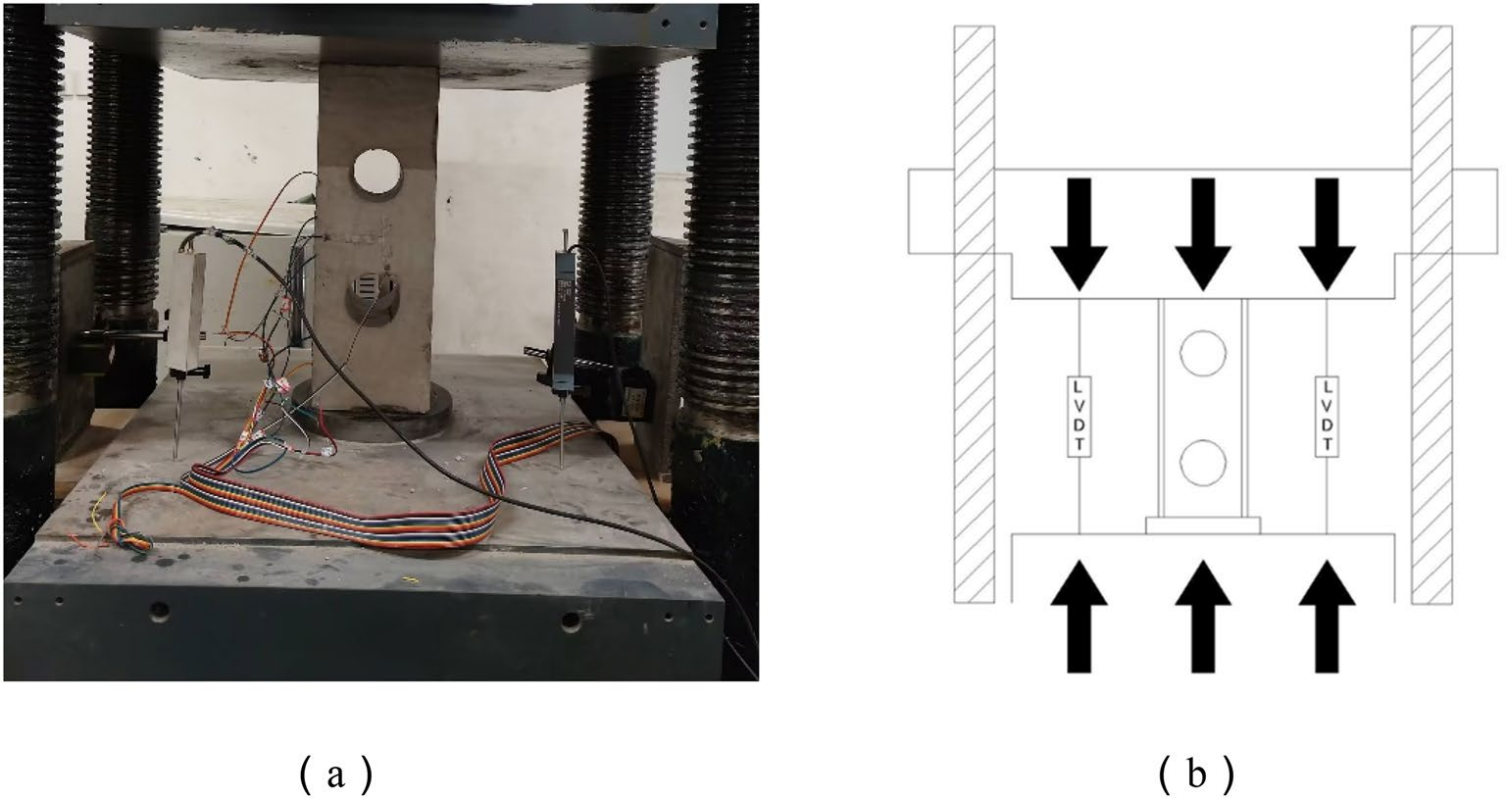
Test loading device. (**a**) Test load device and (**b**) Loading settings

An axial compression test was carried out on a 5000KN press, using the method of displacement-controlled loading procedure, with the speed set to 0.5 mm/min. Loading device was shown in Fig. 6, and the procedure stopped as soon as the load on the specimen had been reduced to about 70% of the ultimate load or when serious deformation of the specimen occurred. The required data and phenomena were recorded and the experiment was concluded by slow unloading.

**Figure 7:**
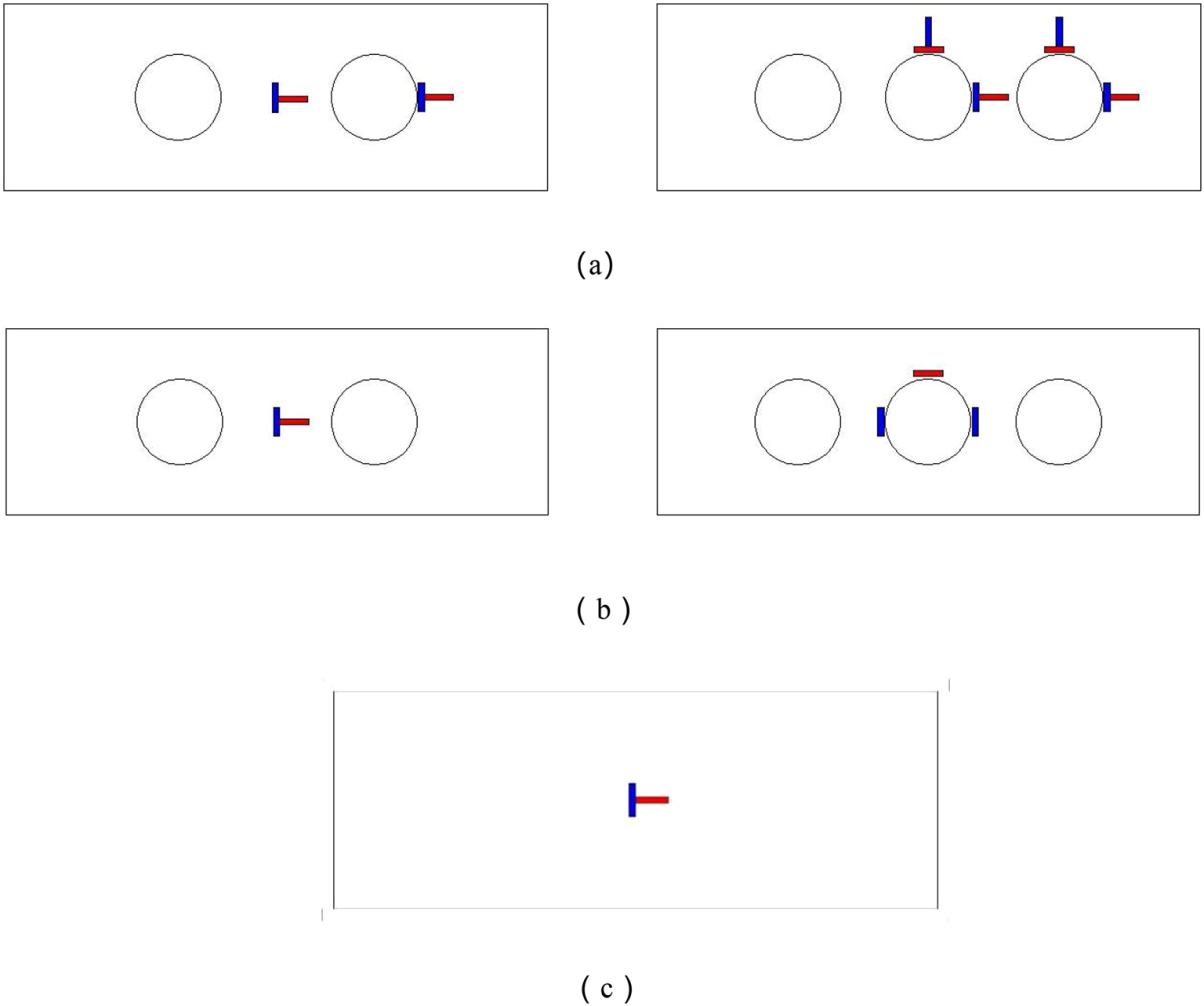
Strain gauge paste position. (**a**) Strain gauge positions for steel profiles,(**b**) Concrete strain gauge placement and (**c**) Flange strain gauge position

As illustrated in Fig. 7b, two displacement gauges of 50 mm range were mounted on the lower bearing plate to measure the axial movement of the member and strain gauges were pasted on the steel and concrete respectively, the specific paste position was shown in Fig. 7, which is used to measure the strain at each position.

## Results and analyses

### Failure modes

**Figure 8:**
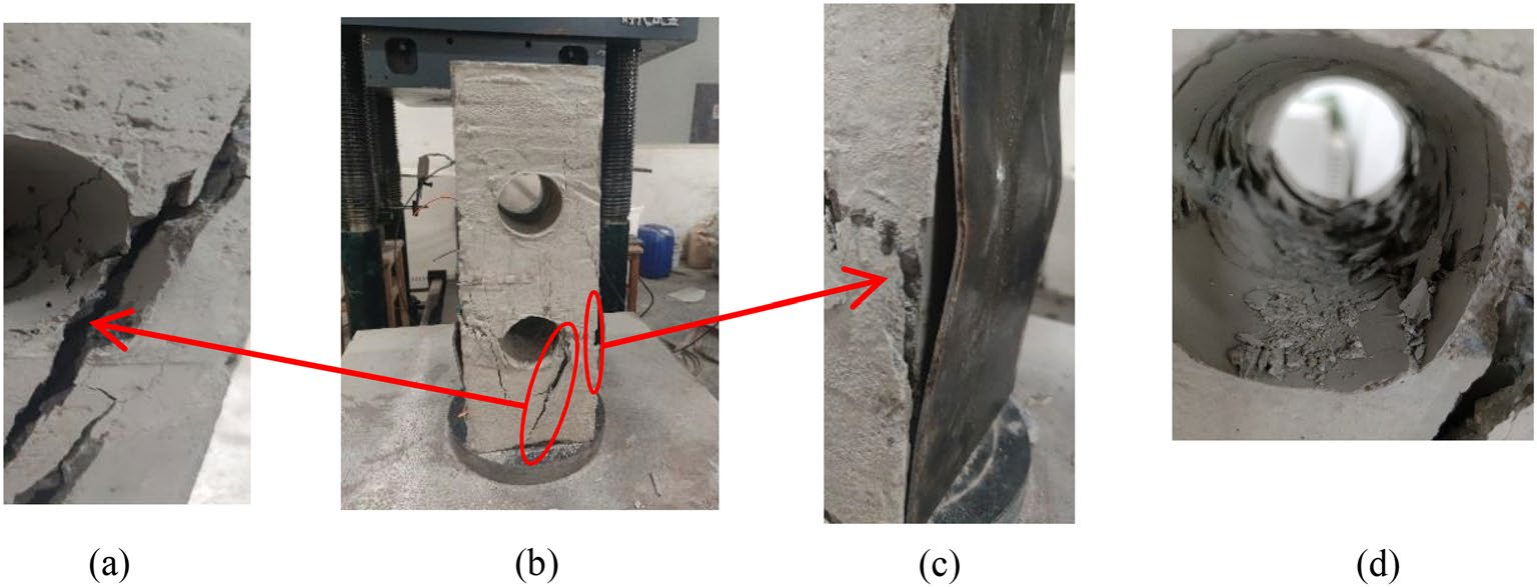
Loading process and phenomena. (**a**) Crack expansion, (**b**) Destruction of compression columns, (**c**) Partial flexion of the flange and (**d**) Concrete falling out of the hole

For the test composite columns, the damage was mainly the breaking of concrete and local buckling of profile flanges, and a similar phenomenon was observed for the PEC columns with web opening, although the damage modes of the individual specimens were relatively close to each other, there were still some gaps between the specimens due to the influence of the parameters such as the opening rate and shape of the open holes. As shown in the Fig. 8b, the typical failure modes observed are as follows:

1. Local buckling of the flange. In axial pressure loading, because the concrete would limit the inward buckling of the flanges, bottom hole near the section of the flange on both sides of the outward sinusoidal half-wave buckling, protruding from the distance of 2–3 mm, leading to the link and the flange broken, the specific situation is shown in Fig. 8c.
2. Concrete crushing. At the beginning of loading, the location of the concrete hole edge would produce several cracks, as the load increased, the cracks progressively widened until the cracks were connected, resulting in concrete stripping and gravel exposuring, as shown in Fig. 8a and at the same time, the hole would produce cracks and accompanied by the fall of concrete fragments, as illustrated in Fig. 8d.

**Figure 9:**
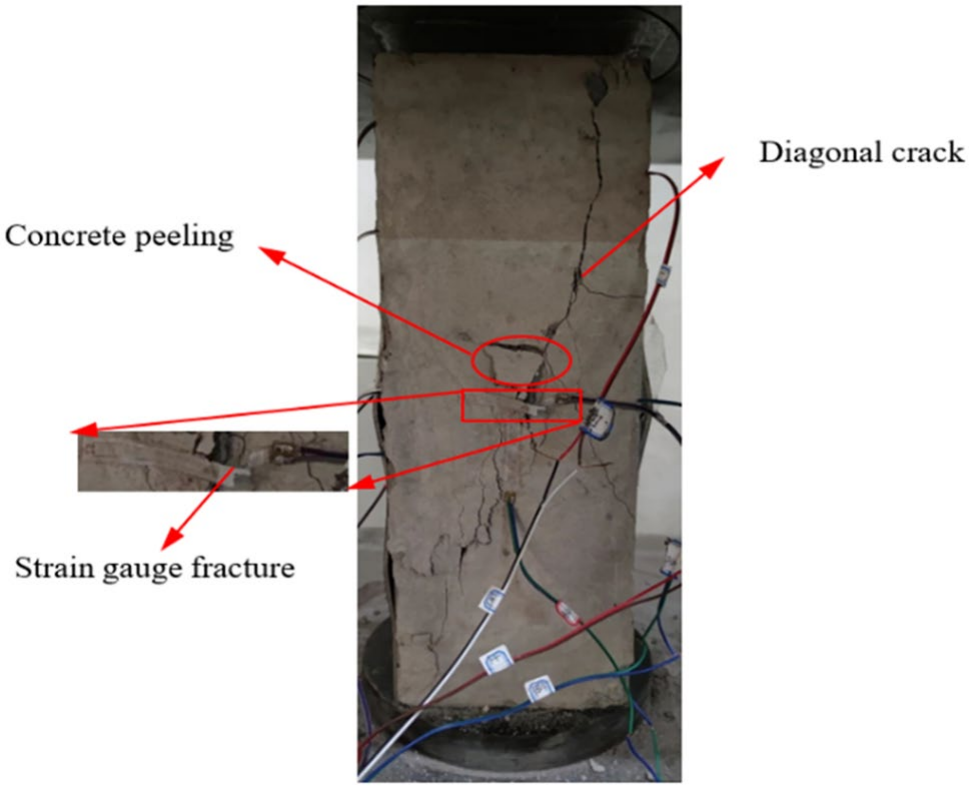
Destruction mode of PEC-30.

As depicted in Fig. 9 the PEC-30 specimens did not show obvious local damage patterns due to the absence of obvious holes. Therefore, as the load increased, transverse cracks or oblique cracks of different lengths appeared on the concrete surface of the specimen, accompanied by peeling and falling of the concrete, and the strain gauges were broken. While the PEC-30 still had a certain degree of integrity after the peak load, and the bearing capacity decreased slowly, and the specimen still had a certain degree of bearing capacity after the applied load was stopped.

### Analysis of factors affecting carrying capacity

**Figure 10:**
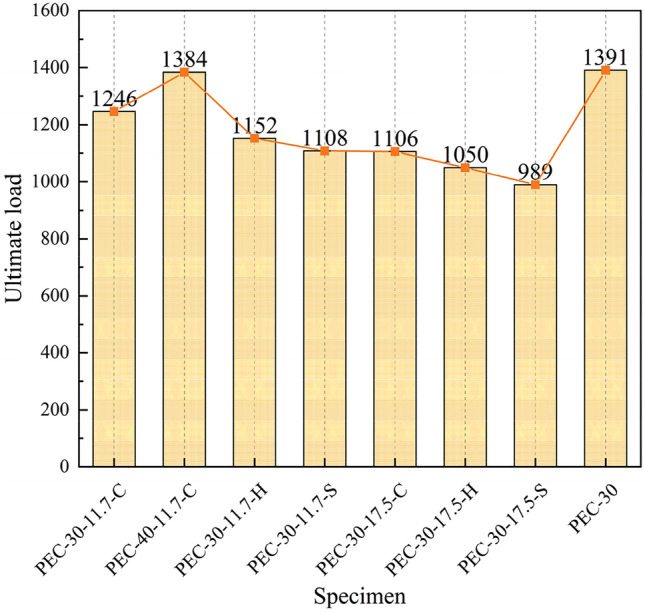
Variation of ultimate load.

To analyze the axial compression performance of PEC columns with web openings by comparing the ultimate load-carrying capacity and ductility, Fig. 10 shows the variation in ultimate load-carrying capacity of the specimens with various shapes of openings at different opening rates.

#### Impact of opening

As is noted in Fig. 10, the load-carrying capacity of the solid web PEC column was 1391 kN, and the PEC-30-11.7-C has a capacity of 1246 kN, a reduction of 10.42%. This was because the open hole in the web would make the member produce stress concentration at the hole location. Conversely, the open hole in the web would result in the deformation of the concrete and steel section being unable to be effectively suppressed, thereby reducing the load-carrying capacity. During the vertical load transfer process, the transfer path was altered in the vicinity of the open hole. The circular hole, devoid of sharp corners, facilitates the transfer of force along the edges of the circular hole, thereby preventing the occurrence of any discernible stress concentration phenomenon.

#### Influence of opening rate

**Figure 11:**
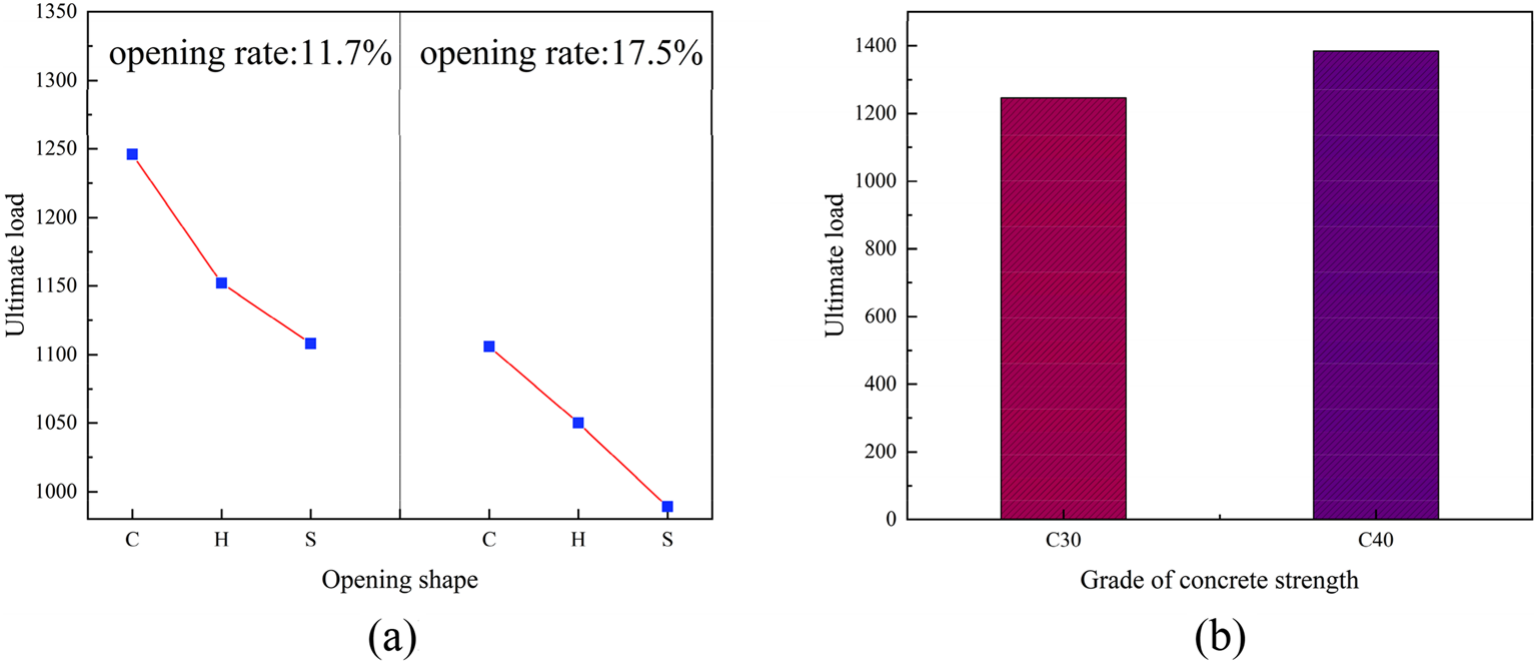
Variation of ultimate load.

As is noted in Fig. 11a the PEC columns with web opening would help in steel cost saving and help in transportation of wires and pipes. The load-carrying capacity of the web open PEC column with 11.7% (2 circular holes) was 1246 kN and web open PEC column with 17.5% (3 circular holes) was 1106 kN. The load-carrying capacity was reduced by 11.2% due to the increase in the rate of opening.

#### Effect of hole shape

The load-carrying capacity of the web opening PEC columns differed according to the shape of the holes (circular, hexagonal, square). The load-carrying capacity of the columns with circular holes was 1246 kN, that of the column with hexagonal holes was 1152 kN, and that of the column with square holes was 1108 kN. The effect of the hole shapes on the load-carrying capacity of the PEC columns with web opening was relatively small. The load-carrying capacity of the columns with circular holes was reduced by 7%. The load-carrying capacity of the columns with hexagonal holes was found to be 5% greater than that of the columns with square holes. Furthermore, the columns with square holes exhibited a 7% reduction in load-carrying capacity compared to those with hexagonal holes. The load-carrying capacity of the PEC column with square holes was found to be 11.1% lower than that of the column with circular holes.

#### Effect of concrete strength

From Fig. 11b, it is evident that the load-carrying capacity of PEC columns with web openings increases with higher concrete strength grades. However, due to the limited synergistic effect between low-strength concrete and high-strength steel, coupled with the higher cost associated with high-strength concrete, the increase in load-carrying capacity of the specimens was constrained. Consequently, columns with web openings were designed using C30 and C40 strength grade concrete, yielding ultimate load capacities of 1246 kN and 1384 kN respectively. In comparison with solid web columns, the load-carrying capacity of the columns with web openings decreased by 10.4% and 0.5%, respectively. Notably, the load-carrying capacity of the column with web opening and C40 strength grade concrete closely approached that of the solid web column.

In essence, the web opening columns exhibited a lack of robust inhibition of concrete as a solid web column due to the presence of holes, which consequently affected the overall performance. The web opening resulted in a reduction in the ultimate load-carrying capacity of the members, which was due to the destruction of the geometrical continuity of the members. As illustrated in Fig. 11a, the ultimate load-carrying capacity of the PEC composite columns with varying openings exhibited a correlation with the shape of the openings. It can be observed that the ultimate load-carrying capacity of the member decreased with an increase in the opening rate, as the greater the number of openings, the fewer steel sections available for collaboration with the concrete. The ultimate load-carrying capacity of the circular, hexagonal, and square holes decreased at two types of opening rate. From Fig. 11b, it is evident that an increase in concrete strength grade led to a 11.08% enhancement in the ultimate load-carrying capacity of the PEC column. This was attributed to the fact that an elevated concrete strength enhanced the crack resistance of the member and the adhesion of the concrete, which in turn compensated for the detrimental effects of the holes on the specimen. As a consequence of the enhancement in concrete grade, the concrete load-carrying capacity was augmented, thereby enhancing the ultimate load-carrying capacity of the column.

### Ductility index

The ductility of web opening PEC columns was analyzed using the ductility index, which is defined as follows^4^:

1 $$ DI = \frac{{\delta_{0.85\% } }}{{\delta_{u} }} $$

where δ0.85% to be the axial displacement when the load is reduced to 85% of the peak load and δ_u_ to be the displacement of the member at peak load.

**Figure 12:**
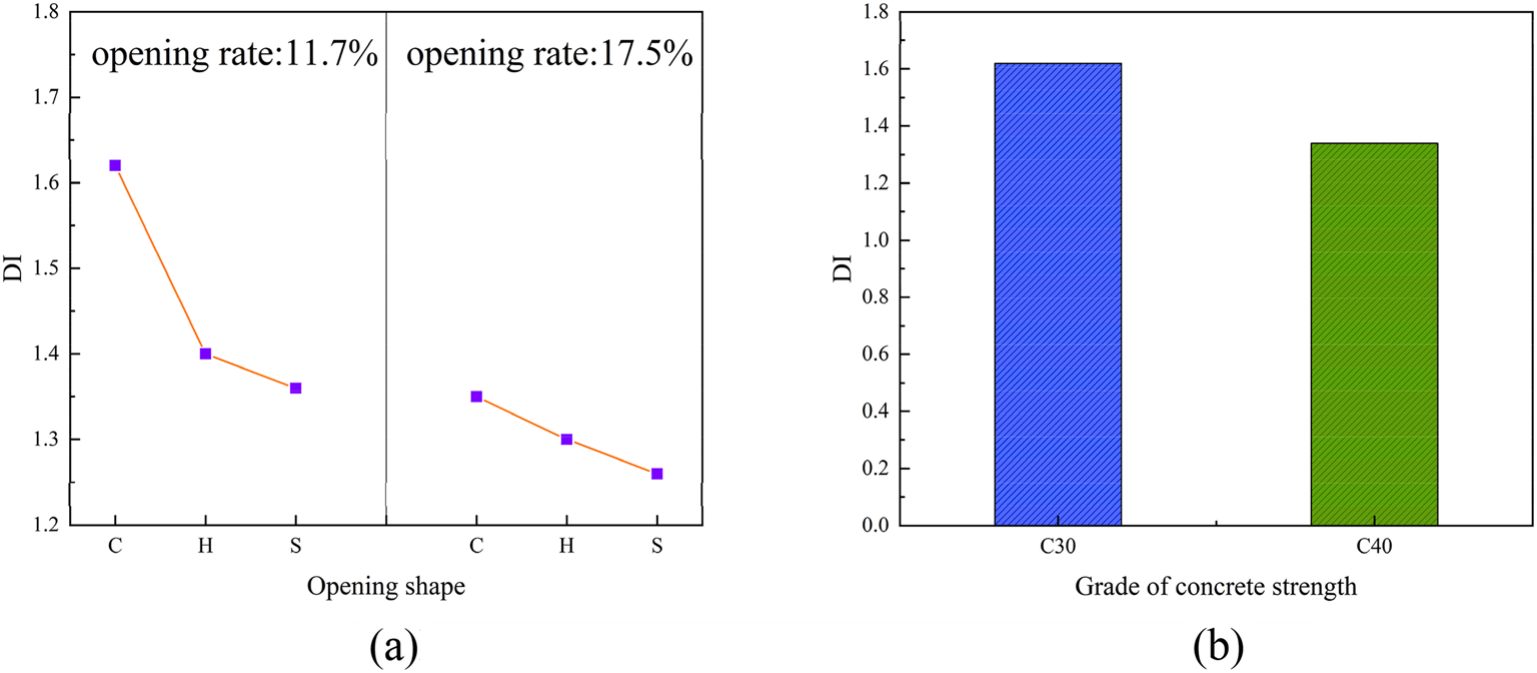
Variation of ductility index.

As depicted in Fig. 12, it is evident that as the opening rate increases, the ductility index decreases. Specifically, the ductility index follows the order of circular holes, hexagonal holes, and square holes, with a decreasing trend. Moreover, the increase in concrete strength grade enhances the ultimate bearing capacity of PEC composite columns. However, it also diminishes the ductility of these columns, resulting in a reduction of 17.3% in ductility.

#### Effect of opening shape on ductility index

The shape of the open holes has a significant impact on the force distribution and stress concentration of the PEC column with web opening, which in turn affects the ductility of the specimen. In contrast, the ductility index of the PEC column without web opening is relatively low, and the opening effectively enhance the ductility index of the specimen. From the Fig. 12a, it can be seen that the circular hole has the greatest ductility index of all the shapes when the opening rate is certain.

#### Effect of opening rate on ductility index

The introduction of higher openings results in a reduction in the effective cross-sectional area of the web, which subsequently affects the overall load-carrying capacity and stability of the column. Consequently, this has an impact on the ductility of the structure. The ductility index of specimens with higher hole openings is found to decrease, with a corresponding decrease in the ductility index of specimens with circular holes of 16.7% when the hole opening ratio increases from 11.7 to 17.5%.

#### Effect of concrete strength grade on ductility index

The strength of the concrete has a direct impact on the overall load-carrying capacity and stability of the PEC with columns web opening. The increase in concrete strength as shown in Fig. 12b decreases the ductility of the corresponding specimen. This is because increasing the strength of concrete is often accompanied by an accelerated hardening process, which results in it becoming more brittle.

### Load–displacement curve

**Figure 13:**
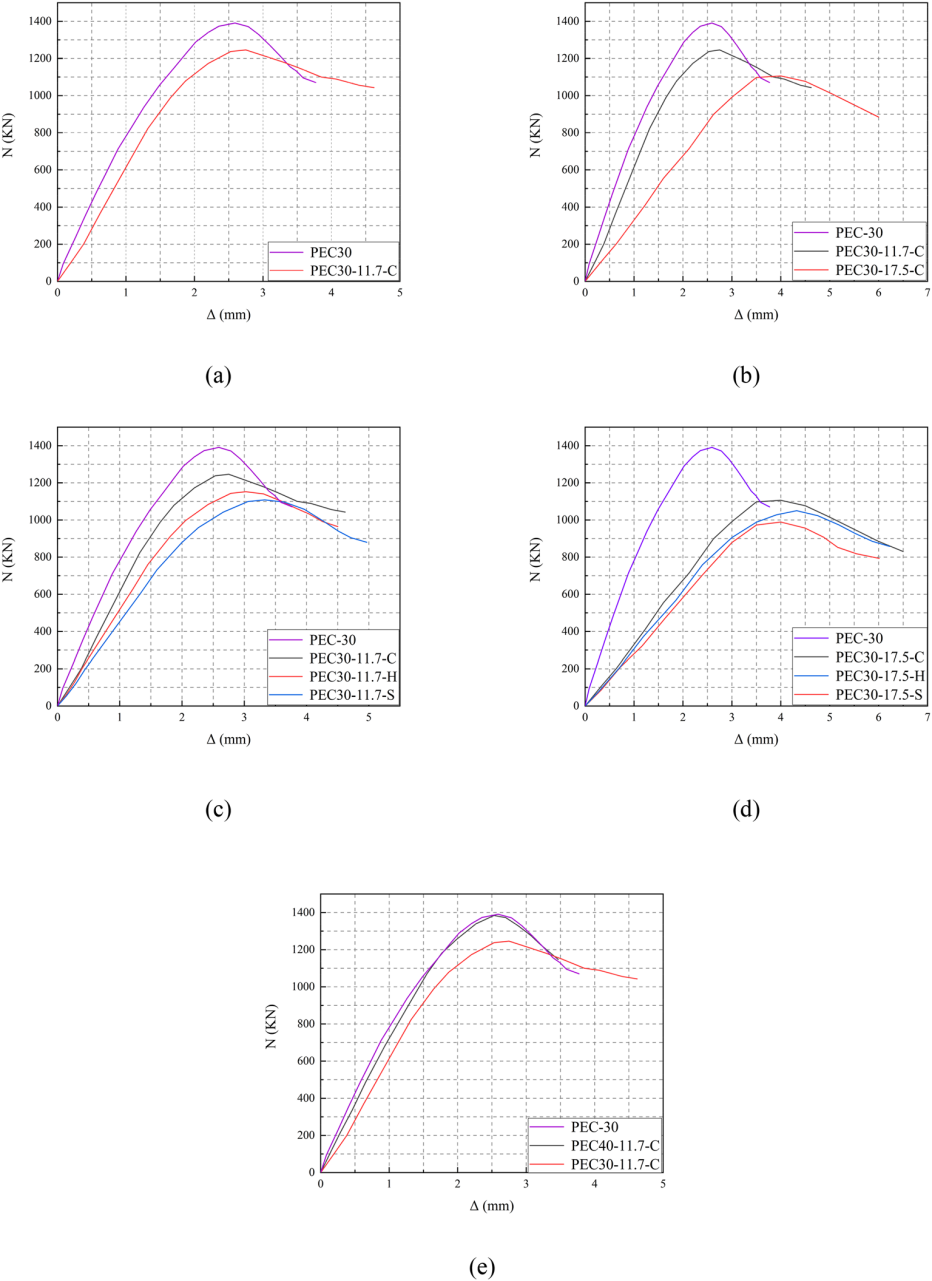
Load–displacement curves for different parameters.

Figure 13a–e shows the effect of opening, opening rate, opening shape, and concrete strength on the load–displacement curves, respectively. The results showed that, as a result of the elastic–plastic properties of the material. The curves of the members showed a linear increase, a nonlinear decrease and finally a tendency to stabilize, which correspond to the three stages of the axial compression of the members: elastic working stage, elastic–plastic working stage, and destructive stage, respectively. After the curve reached to the peak load, most of the concrete had been damaged and lost its load-bearing capacity. So the curve decreased as the displacement increased, the load changed slowly, the main load was borne by the column gradually began to flex outward, and the curve tended to stabilize.

#### Presence of holes

As Fig. 13a shows the comparison of the load–displacement curves before and after the opening of the PEC columns. It can be seen from the figure that the opening reduces the stiffness of the PEC columns, making them more susceptible to deformation when subjected to forces, leading to a reduction in the stability of the overall structure. In the descending phase of the curve, the rate of decline of the specimen before opening the hole is faster than that after opening the hole, which is due to the fact that opening the hole increases the ductility of the specimen.

#### Opening rate

As depicted in Fig. 13b, this illustration compares load–displacement curves at various opening rates. As the opening rate increases, the effective cross-sectional area of the specimen diminishes, resulting in a reduction in the specimen's overall stiffness. In the elastic phase, there is a linear correlation between load and displacement, with the slope reflecting the specimen's stiffness. Consequently, an increase in the opening rate will result in a decline in the specimen's stiffness, which will in turn lead to a reduction in the slope of the load–displacement curve in the elastic phase. Furthermore, in the downward phase of the curve, the curve with a higher open rate will decline at a faster rate compared to the curve with a lower open rate.

#### Opening shape

The effects of different opening shapes on the load–displacement curves are illustrated in Fig. 13c,d. The impact of opening shapes on load–displacement curves is relatively minor. It can be observed that circular and square-hexagonal openings are effective in reducing stress concentration and delaying the yielding of composite columns. In contrast, square openings have the opposite effect, exacerbating stress concentration and leading to a more pronounced yield behavior. This results in the specimen with square openings producing more cracks and exhibiting a faster rate of descent in the falling phase.

#### Concrete strength grade

Figure 13e illustrates the impact of concrete strength on the load–displacement curve. Concrete of greater strength typically exhibits a higher modulus of elasticity, which results in PEC columns constructed from high-strength concrete demonstrating enhanced stiffness when subjected to loads. Consequently, the load–displacement curves may exhibit greater slope during the elastic phase. Nevertheless, high-strength concrete is also frequently more brittle. Consequently, PEC columns constructed from high-strength concrete may demonstrate a more rapid decline in load during the subsequent decline phase. This is due to the fact that cracks tend to develop more rapidly when high-strength concrete reaches its ultimate strength, resulting in a rapid decline in load.

## Simplified design method

Considering the load-bearing capacity of the joint action of profile and concrete, the calculation of the bearing capacity of the PEC column is carried out by using the superposition method, where the carrying capacity of the concrete is added to the carrying capacity of the profile by the following formulae^26^:

2 $$ N_{u} = 0.85A_{C} f_{C} + A_{a} f_{a} $$

where 0.85 is the strength discount factor, ${A}_{C}$ is the concrete cross-sectional area, ${f}_{C}$ is concrete axial compressive strength, ${A}_{a}$ is the section area of the steel section, ${f}_{a}$ is the yield strength of the steel section, ${f}_{c}=0.67{f}_{cu}$, where ${f}_{cu}$ is the concrete cubic compressive strength, *N*_u_ is the load-carrying capacity of PEC column without web opening;

For the link, the load carrying-capacity is as Eq. (3)^27^

3 $$ P_{sv} = 0.43A_{sv} \sqrt {f_{c} E}_{c} $$

where $P_{sv}$ is the load carrying-capacity provided by a single link, $A_{sv}$ is the cross sectional area of the link, $f_{c}$ is concrete axial compressive strength, $E_{s}$ is the modulus of elasticity of the concrete.

**Figure 14:**
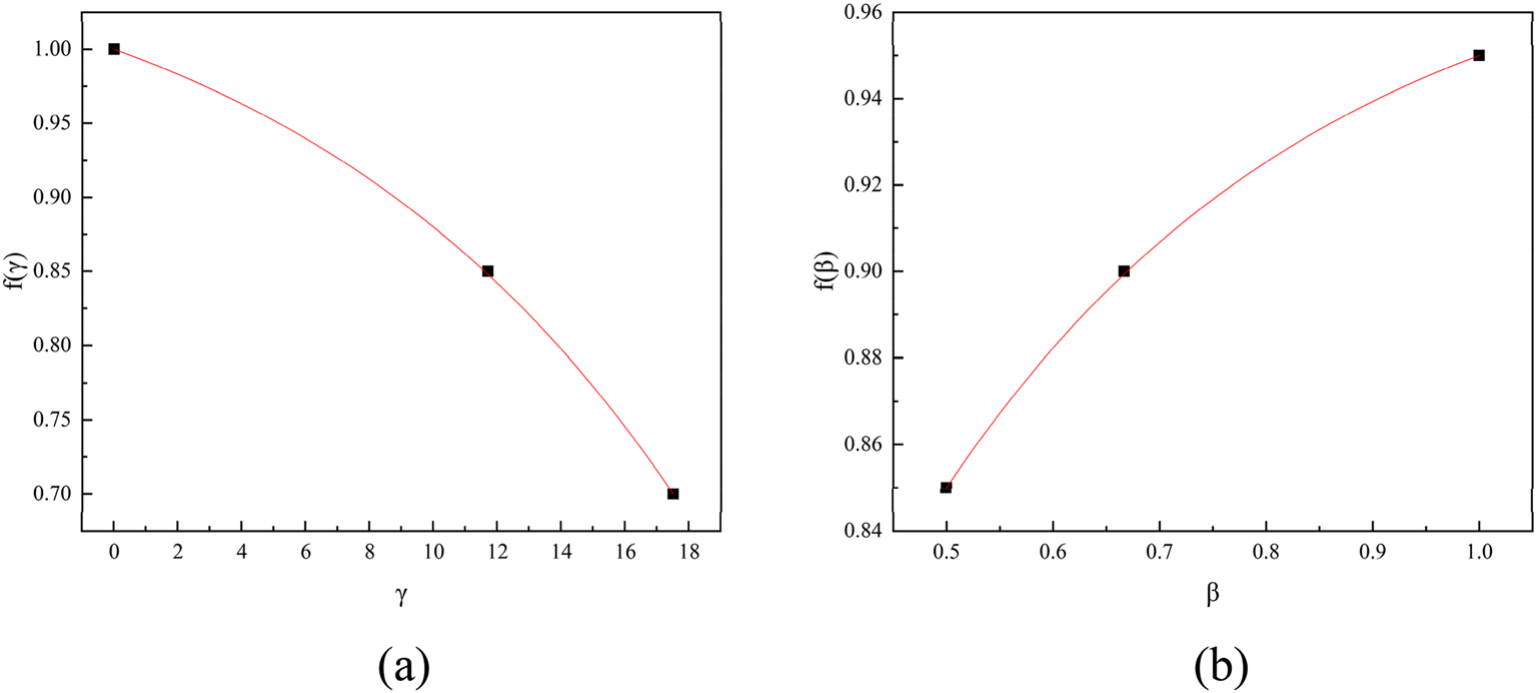
Regression curves with coefficients.

In Liu's experiments^28^, the integral method was used to discount the web-opening PEC members, while in the present experiments the discount factor was improved and the load-bearing capacity of each part was calculated separately. Based on the experimental data, the opening rate reduction factor $f({\upgamma })$, the opening shape factor reduction factor $f({\upbeta })$ were fitted as follows, finally the curves are shown in Fig. 14.

4 $$ f(\gamma ) = 1.098 - 0.98e^{{\frac{\gamma }{12.47}}} $$

5 $$ f(\beta ) = - 0.53e^{{\frac{ - \beta }{{0.36}}}} + 0.984 $$

where $\gamma$ is the opening rate, depending on the number of internal angles of each opening shape, $\beta$ is related as Eq. (6).

6 $$ \beta = \frac{n - 2}{n},n \ge 3 $$

where $f({\upbeta })$ is the opening shape discount factor, $f({\upgamma })$ is the opening rate discount factor.

The final formula for the axial compressive load capacity of partially encased concrete columns with web opening was derived as Eq. (7). The calculation results are shown in Table 4.

7 $$ N_{t} = f({\upgamma })f({\upbeta })(0.85A_{c} f_{{\text{c}}} + A_{a} f_{a} ) + \sum {P_{sv} } $$

**Table 4:** Comparison of test data and the calculation results.

Specimen	Section size H × B × t_w_ × t_f_	Experimental value ${N}_{e}$/kN	calculated value ${N}_{t}$/kN	$\frac{{N}_{t}}{{N}_{e}}$
PEC-30–11.7-C	180 × 180 × 4 × 4	1246	1303	1.05
PEC-30–11.7-H	180 × 180 × 4 × 4	1152	1245	1.08
PEC-30–11.7-S	180 × 180 × 4 × 4	1108	1187	1.07
PEC-30–17.5-C	180 × 180 × 4 × 4	1106	1174	1.06
PEC-30–17.5-S	180 × 180 × 4 × 4	989	1078	1.09
PEC-30–17.5-H	180 × 180 × 4 × 4	1050	1126	1.07
PEC-30	180 × 180 × 4 × 4	1391	1567	1.12

## Conclusion

Within this article, in comparison to the conventional structural form, the PEC column with web opening exhibits a combination of the advantageous properties of steel and concrete, and enhances the performance of the structure through the opening design. By conducting a comprehensive investigation into the impact of varying opening parameters on column performance and proposing novel opening design parameters, this study introduces novel insights and methodologies for research and application in this field. Focusing on the effect of opening shape, opening rate, concrete strength and other factors on the columns. The study had come to the below conclusions:

1. The damage modes of the PEC columns with web opening were the crushing of concrete and local buckling of the flange, accompanied by the generation of cracks in the holes and the falling of concrete fragments.
2. Although the web opening reduced the load-carrying capacity of the column due to the stress concentration phenomenon, it saved steel to a certain extent and would have a certain development prospect in the future.
3. The load-carrying capacity of the columns with circular holes in the web was slightly lower compared to that of the solid web column, which was within acceptable limits, whereas the hexagonal holes and square holes reduced the load-carrying capacity by a relatively large amount.
4. As the opening rate increased, the ultimate load-carrying capacity of the columns gradually decreased. Two circular holes in the web of the member column with an 11.7% opening rate had the best mechanical performance.
5. The superposition method was adopted to compute the ultimate load-carrying capacity of PEC composite columns with web opening, besides the web opening discount factor and the opening shape discount factor were implemented to calculate the ultimate load-carrying capacity.
6. The objective of this study is to examine the impact of various opening shapes, sizes, and positions on the performance of the PEC columns with web opening. The goal is to identify the optimal design parameters for openings through a combination of experimental investigations and numerical simulations.
7. Further research into the fire resistance of the PEC column with web opening is warranted to explore the influence of these openings on column stability and durability in fire scenarios. Additionally, potential applications of refractory materials and green concrete should be investigated.

## Data Availability

Data is provided within the manuscript.
